# Cost and affordability of healthy diets in Vietnam

**DOI:** 10.1017/S1368980023002665

**Published:** 2023-12-01

**Authors:** Duong TT Van, Anna W Herforth, Huong T Trinh, Binh TT Dao, Ha TP Do, Elise F Talsma, Edith JM Feskens

**Affiliations:** 1 Division of Human Nutrition and Health, Wageningen University & Research, Wageningen, The Netherlands; 2 Department of Nutrition and Food Science, University of Medicine and Pharmacy, Ho Chi Minh City, Vietnam; 3 Department of Global Health and Population, Harvard T.H. Chan School of Public Health, Harvard University, Boston, MA, USA; 4 Department of Mathematics and Statistics, Thuongmai University, Ha Noi, Vietnam; 5 Department of Economics, Hanoi University, Ha Noi, Vietnam; 6 National Institute of Nutrition, Ministry of Health, Hanoi, Vietnam

**Keywords:** Cost of a healthy diet, Affordability, Seasonality, Food-based dietary guidelines

## Abstract

**Objectives:**

To estimate the cost and affordability of healthy diets recommended by the 2016–2020 Vietnamese food-based dietary guidelines (FBDG).

**Design:**

Cross-sectional analysis. The Cost of a Healthy Diet (CoHD) indicator was used to estimate the lowest cost of healthy diets and compare the cost differences by food group, region and seasonality. The affordability of healthy diets was measured by further comparing the CoHD to food expenditures and incomes.

**Setting:**

Food prices of 176 food items from January 2016 to December 2020 were derived using data from monthly Consumer Price Index databases nationally and regionally.

**Participants:**

Food expenditures and incomes of participants from three latest Vietnam Household Living Standard Surveys were used.

**Results:**

The average CoHD between 2016 and 2020 in Vietnam was 3·08 international dollars using 2017 Purchasing Power Parity (24 070 Vietnamese Dongs). The nutrient-rich food groups, including protein-rich foods, vegetables, fruits and dairy, comprised approximately 80 % of the total CoHD in all regions, with dairy accounting for the largest proportion. Between 2016 and 2020, the cheapest form of a healthy diet was affordable for all high-income and upper-middle-income households but unaffordable for approximately 70 % of low-income households, where adherence to the Vietnamese FBDG can cost up to 70 % of their income.

**Conclusions:**

Interventions in local food systems must be implemented to reduce the cost of nutrient-rich foods to support the attainment of healthier diets in the Vietnamese population, especially for low-income households.

Poor diet quality plays a central role in morbidity and mortality worldwide due to both insufficient intake of healthy foods and excessive intake of unhealthy foods^(1)^. The cost and affordability of healthy diets are among the essential drivers of food choices, diet quality, and nutrition outcomes^(2,3)^ and are among the biggest challenges to food security^(4)^. For some individuals, access to sufficient dietary energy is also a challenge, let alone the access to healthy diets. A growing body of evidence shows that a healthy diet is more expensive than an unhealthy diet^(5)^. Nutrient-rich foods, such as fruits, vegetables, protein-rich foods and dairy, account for a much higher proportion of the total cost of a healthy diet than energy-dense foods (i.e. starchy staples)^(4)^.

As higher relative costs can directly affect the consumption of nutrient-rich foods, a shift to healthier diets requires these foods to be available and affordable, especially for poor populations^(6)^. The poor tend to be more sensitive to food prices and the impact of food prices on demand for food is greatest among the poorest people^(7)^; consequently, they face more barriers in affording healthy diets and improving their diet quality^(8)^. Furthermore, the cost and affordability of healthy diets are significantly variable across food groups, geographical zones, seasonal differences and local food systems. For example, the higher perishability of fruits, vegetables and animal-source foods often result in higher prices as they are more dependent on production and food supply chain efficiency at the local level^(3)^. Therefore, conducting country-specific analyses of the cost and affordability of healthy diets can offer the insights required to perform local interventions and systemic innovations in the food system, making healthier diets more accessible for poorer populations^(9)^.

In Vietnam, achieving food security requires special attention to access to healthy diets foods for all population groups. Our previous work showed that Vietnamese households with lower incomes also have lower diet quality^(10)^. Food-based dietary guidelines (FBDG) are state-published definitions of the healthy diets appropriate for the population^(11)^, and adherence to FBDG can be used to measure diet quality^(10)^. Despite this, no studies have assessed whether the Vietnamese FBDG are feasible and affordable for all. If certain subpopulations cannot adhere to the FBDG due to lower incomes, their diet quality will remain limited. To this end, reducing food costs will facilitate higher adherence to FBDG and, therefore, increase the diet quality of the population.

Thus, the aims of the present study were: (1) to estimate the minimum cost of meeting the healthy diets recommended by the 2016–2020 Vietnamese FBDG and to compare the differences in the costs by food group and by region; (2) to examine the seasonality in the cost of healthy diets; and (3) to assess the affordability of healthy diets in Vietnam. Achieving these outcomes will provide information on the cost of specific components of a healthy diet, such as what (i.e. food group), when (i.e. seasonality), where (i.e. region) and to whom (i.e. low-income households) food must be made more affordable. This, in turn, can facilitate interventions to support local systems in ensuring the affordability of healthy diets and increase the possibility of achieving higher diet quality in the Vietnamese population.

## Data and methods

### Consumer Price Index food price data

In this study, we used the average monthly price of food items in the list of food and non-alcoholic beverages collected across sixty-three provinces by the General Statistics Office (GSO) for the monthly Consumer Price Index (CPI). We obtained price data of 176 food items at the national level (average of sixty-three provinces) and regional level (data of twenty-five provinces representing six geographical regions) from January 2016 to December 2020 (see online supplementary material, Supplemental Table S1). We used CPI data to calculate the cost of healthy diets because it allows us to look at the trend in cost and affordability over time. The monthly CPI food price database is well represented at both national and regional levels, allowing us to estimate regional spatial and seasonal variations.

### Vietnam Household Living Standard Survey

The Vietnam Household Living Standard Survey (VHLSS) data are to calculate mean daily food expenditure and daily per capita income. This survey has been conducted by the GSO with technical support of the World Bank every 2 years since 2002. Each VHLSS wave is made of two sub-surveys, including household sub-survey and commune sub-survey. The households in each VHLSS survey are selected by a two-stage area sample design where communes are selected in the first stage, and three enumeration areas per commune are selected in the second stage. The sampling procedure of this survey has been described in more detail elsewhere^(12–14)^. We used data from three latest surveys at the household sub-survey level, including VHLSS 2016 (9399 households), VHLSS 2018 (9167 households) and VHLSS 2020 (9388 households) to calculate mean daily food expenditure and daily per capita income. The methodology for calculating these indicators has been described in more detail elsewhere^(12–14)^.

### Inflation adjustment and currency conversion

We adjusted the nominal values of both CPI food price data and VHLSS data for monthly inflation rates from 2016 to 2020 using the CPI. We collected monthly CPI for food items ( 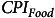 ) from the GSO, Vietnam, and adjusted the food prices to a base of reference period at which 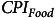 = 100^(15)^. Then we converted local currency unit Vietnamese Dong (VND) into International Dollar ($) using the most recent PPP exchange rates published by the World Bank^(16)^ and not market exchange rates. A reference period of December 2017 was applied for both inflation adjustment and currency conversion. Thus, the cost of healthy diets, food expenditure and income data are reported in 2017 PPP$ and simply presented as $ in the text.

### The cost of healthy diets measurement

In the present study, healthy diets are defined based on the 2016–2020 Vietnamese FBDG for adults, as described in more detail below. We estimated the cost of healthy diets using the Cost of a Healthy Diet (CoHD), which is a metric of least-cost diet that meets FBDG, based on food group classifications. The metric was developed by Herforth *et al.* ^(17,18)^ and has been used in a global analysis^(18)^ and in several countries in Asia^(19–21)^. To calculate the CoHD, the 1–3 lowest cost items were selected in each food group, and then the average prices of the least-cost items were applied to recommended intakes found in the FBDG to arrive at cost estimates.

#### 2016–2020 Vietnamese food-based dietary guidelines

The 2016–2020 Vietnamese FBDG recommend daily consumption of eight food groups, including grains, protein-rich foods, vegetables, fruits, dairy, fats and oils, sugar and sweets, and salt and sauces. In Table 1, the definition of servings of each food group and the foods to be included in in each food group are derived from the graphic presentation (see online supplementary material, Supplemental Fig. 2.S1, Chapter 2), the official background document of the 2016–2020 Vietnamese FBDG (written and published in Vietnamese)^(22)^ and information provided by the National Institute of Nutrition, Ministry of Health, Vietnam.

**Table 1:** Composition of a healthy diet recommended by the 2016–2020 Vietnamese food-based dietary guidelines for adults

Food groups	Main food items	Recommended servings daily	Definition of one serving
Grains	Rice, bread, noodles, potato, maize, casava	12–15	Provides 20 g carbohydrate
Protein-rich foods	Red meat, poultry, seafood, egg, legumes and beans, soya products	5–6	Provides 7 g protein
Fats and oils	Animal fats, plant-based oil, butter, nuts and seeds	5–6	Provides 5 g total fat
Dairy	Milk, yogurt, cheese	3–4	Provides 100 mg Ca
Vegetables	All type of vegetables, mushroom	3–4	Eighty grams of edible vegetables
Fruits	All type of fruits	3	Eighty grams of edible fruits
Sugar and sweets	Sugar, sweets, honey	< 5	Provides 5 g free sugar
Salt and sauces	Salt, seasoning, sauce	< 1	Provides 5 g salt

#### The Cost of a Healthy Diet calculation

The steps followed in the construction of CoHD are described below:

- Step 1: One hundred and seventy-six food items in the original CPI food price dataset were classified into eight food groups based on the recommendations in the 2016–2020 Vietnamese FBDG. We excluded foods in ‘sugar and sweets’ and ‘salt and sauces’ groups, foods that are not recommended for a healthy diet such as trans fats, processed meats. foods that are non-energetic, ingredients, condiments, baby foods, tea, coffee, alcoholic beverages, and foods with an unclear composition. In the case of multiple types of the same food, only the item with the lowest cost was retained (e.g. in the case of rice, Xi Deo rice and Tam Thom rice were both classified simply as rice, and the more expensive item was dropped). These exclusions resulted in a final CPI food price dataset of eighty-eight food items for CoHD calculation. The number of food items for each food group in both the original CPI food price dataset and final CPI food price dataset is shown in online supplementary material, Supplemental Table S2.
- Step 2: The price unit of each food item in the final CPI food price dataset was standardised to price per gram. For the food items that were in non-standard units such as maize and eggs (given in price per ten items), estimates of the standard weight of these items were employed.
- Step 3: All the food items were matched with the global database to obtain the food composition information for edible portion, energy intake and nutrient content (protein, carbohydrate, total fat and Ca). This global database primarily uses food composition information from the United States Department of Agriculture Food Data Central 2020^(23)^.
- Step 4: The price for each food item was converted into price per edible serving (ref FPN tools):

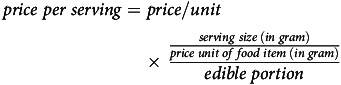

  For the food groups that have a range of servings, the recommended servings were calculated as the average number of servings.
- Step 5: The two cheapest food items for grains, protein-rich foods, and fruits; the three cheapest food items for vegetables; and one cheapest food item for oils and fats and dairy were selected for the CoHD basket^(18)^. The recommended number of servings for each group was multiplied by the average price per serving for each food group to generate the cost of that food group. Finally, the costs of all food groups were summed to obtain the total CoHD.

#### Seasonality measurement

We used seasonal-trend decomposition (STL) as a seasonal adjustment method developed by Cleveland *et al.* ^(24)^. This model decomposes time series into seasonal, trend and remainder components using a filtering algorithm based on the local regression (LOESS). STL assumes an additive relationship between the seasonal, trend and remainder components as follow:

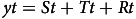




where *y*_*t*_ is the value of the time series at point *t*, *S*_*t*_ is the seasonal component at point *t*, *T*_*t*_ is trend cycle at point *t* and *R*_*t*_ is remainder component at point *t*. The STL algorithm performs smoothing on the time series using LOESS regression in two loops: the inner loop iterates between seasonal and trend smoothing and the outer loop minimises the effect of outliers, as further described in detail elsewhere^(24)^. We used STL with monthly seasonality to decompose time series of total CoHD and CoHD by region and food group from January 2016 to December 2020 to determine seasonality effects. All seasonality models were run in EViews 10.0.

#### Affordability measurement

To assess affordability of healthy diets, we used the indicators described by Herforth *et al.* ^(18)^ and found in the Food Prices for Nutrition DataHub^(25)^. These included the percent of Vietnamese who cannot afford healthy diets across the region and income level between 2016 and 2020 in terms of (1) CoHD compared with the mean daily per capita income that could be spent on food, and (2) the CoHD as share of total food expenditures. For example, food expenditures account for 21 %, 28 %, 32 %, 29 % and 50 % of income on average in high-, upper-middle-, middle-, lower-middle- and low-income households in 2020, respectively (see online supplementary material, Supplemental Table S3). We multiplied these proportions by individuals’ daily income in 2020 to end up with the daily amounts that could be spent on food by each income segment and compared it with the CoHD (they cannot afford healthy diets if these amounts less than the CoHD). Similar calculations are applied for 2016 and 2018. Daily food expenditure was calculated from the VHLSS data per adult female equivalent (AFE) per d. Using the AFE as a reference, the household food consumption was transformed into the intake of a reference individual as a proportion of energy requirements of a non-pregnant, non-lactating woman (aged 20–30 years) based on the recommendations of the Human Energy Requirements, considering age and gender^(26)^. AFE values of other household members were calculated by dividing their energy requirement by the energy requirement of the reference AFE per d, and they were then summed up to obtain the total household AFE.

## Results

### The cost of healthy diets

After inflation adjustment, the average CoHD from 2016 to 2020 in Vietnam was $3·08 in international dollars 2017 PPP (24 070 VND). Over this period, the CoHD was lowest in 2019 at $3·02 (23 559 VND) and highest in 2017 at $3·17 (24 788 VND) per d. The CoHD in 2016, 2018 and 2020 were $3·04 (23 755 VND), $3·08 (24 063 VND) and $3·10 (24 186 VND) per d, respectively (data not shown).

**Fig. 1:**
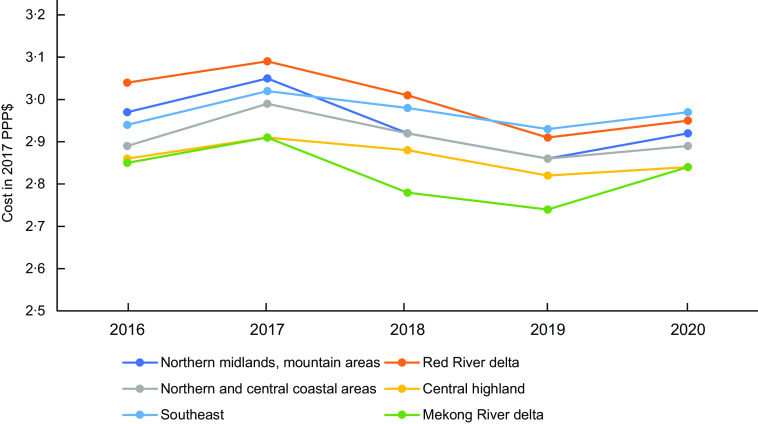
The regional average cost of a healthy diet in 2017 international dollar for an adult per d from 2016 to 2020

The regional average CoHD for an adult per d from 2016 to 2020 is presented in Fig. 1. Among the six regions, the average CoHD was the highest in the Red River delta and the lowest in the Mekong River delta. Although differences between the regions existed, they showed a similar trend over time; all increased from 2016 to reach a peak in 2017, decreased from 2017 to 2019 and then increased again in 2020.

**Fig. 2:**
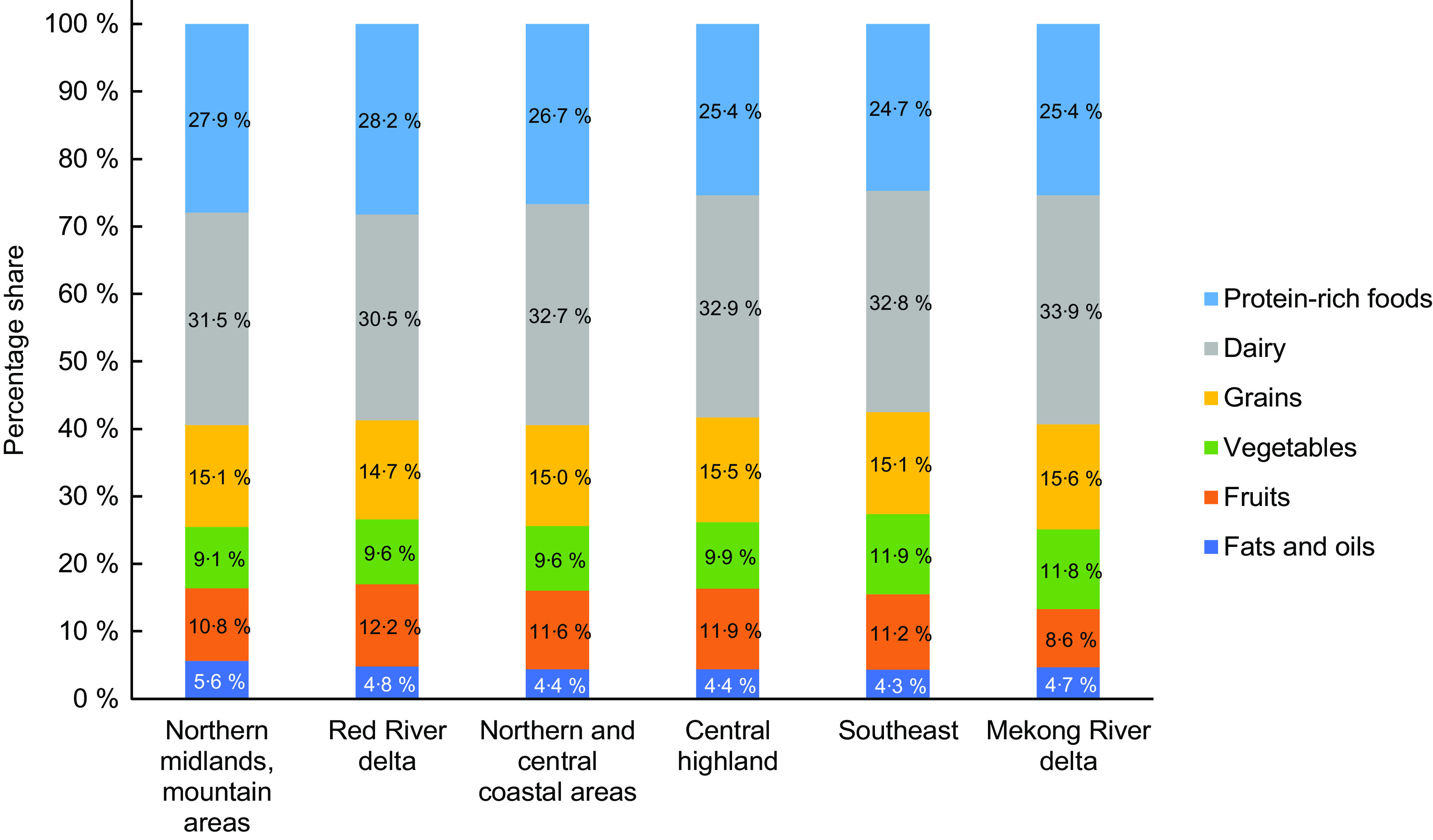
Cost contribution (percentage share of total cost) of each food group in a healthy diet by region (average data from 2016 to 2020)

The protein-rich foods, fruits, vegetables and dairy comprised approximately 80 % of the total CoHD in all regions. Dairy contributed the largest portion of the total CoHD, and fats and oils contributed the least in all regions. The smallest variation in the cost share by food group was observed in fats and oils with a 1·0 % difference between regions, while the largest was in fruits with a 3·2 % difference. The cost share of vegetables was highest in the Southeast (11·9 %) and lowest in the Northern midlands and mountain areas (9·1 %). The cost share of protein-rich foods was highest in the Red River delta (28·2 %) and was lowest in the Southeast (24·7 %). The cost share of grains was comparable among regions at about 15·0 % (Fig. 2).

### Seasonality in the cost of healthy diets

**Fig. 3:**
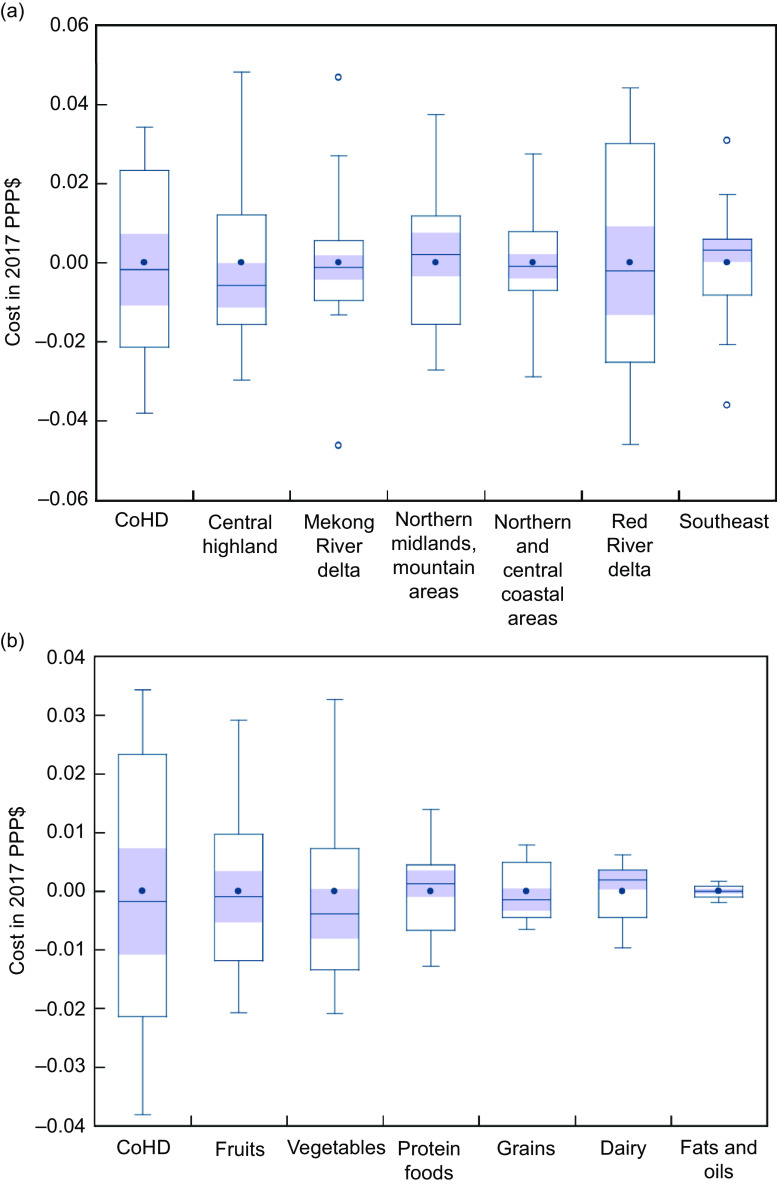
Seasonal variation (in 2017 international dollars) in cost of a healthy diet by (a) food group and (b) region. The size of the box shows the IQR. The bottom and top rules illustrate the fifth and fifty-nine percentiles, respectively. The horizontal bar rule inside the box is the median value for the region or food group

**Fig. 4:**
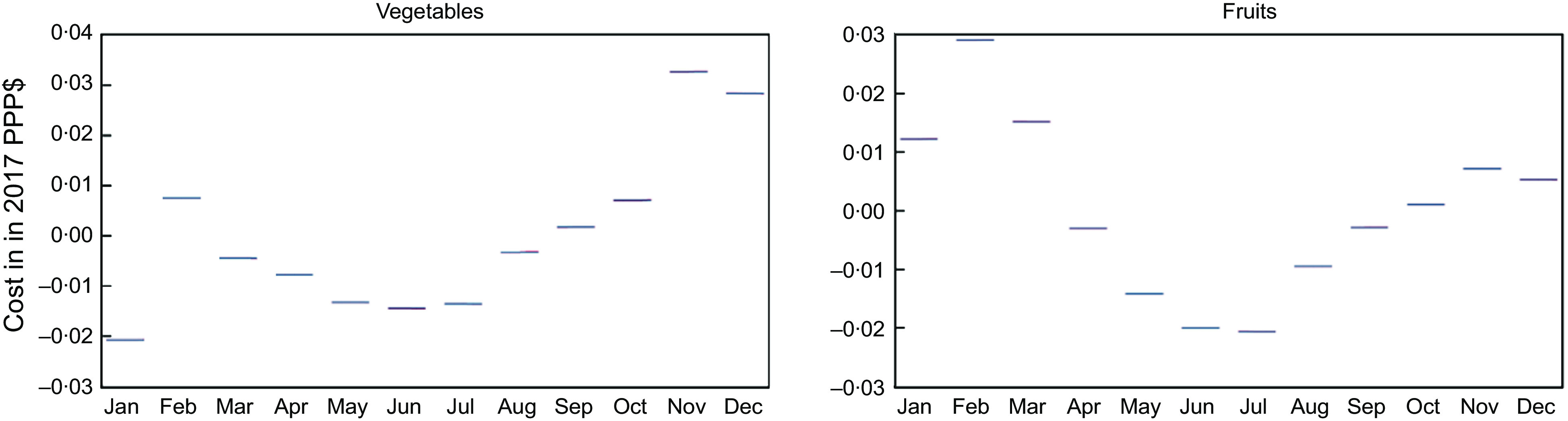
Seasonal variation in cost (in 2017 international dollars) of (a) vegetables and (b) fruits

The seasonal variation in the CoHD by region showed the highest in the Red River delta, followed by the Central highland; the lowest showed in the Southeast and the Mekong River delta (Fig. 3(b)). Across the food groups made up for a healthy diet, a significant seasonal effect in the cost was observed only for vegetables and fruits but not for other food groups such as grains, protein-rich foods, fats and oils, and dairy (Fig. 3(a)). The seasonal variation in cost of vegetables and fruits was likely to be lower in the hot-humid season (from May to August) and higher in the cold-humid season (from November to February); however, the seasonal variation in cost of vegetables dropped in January before increasing in February (Fig. 4).

### Affordability of healthy diets

**Fig. 5:**
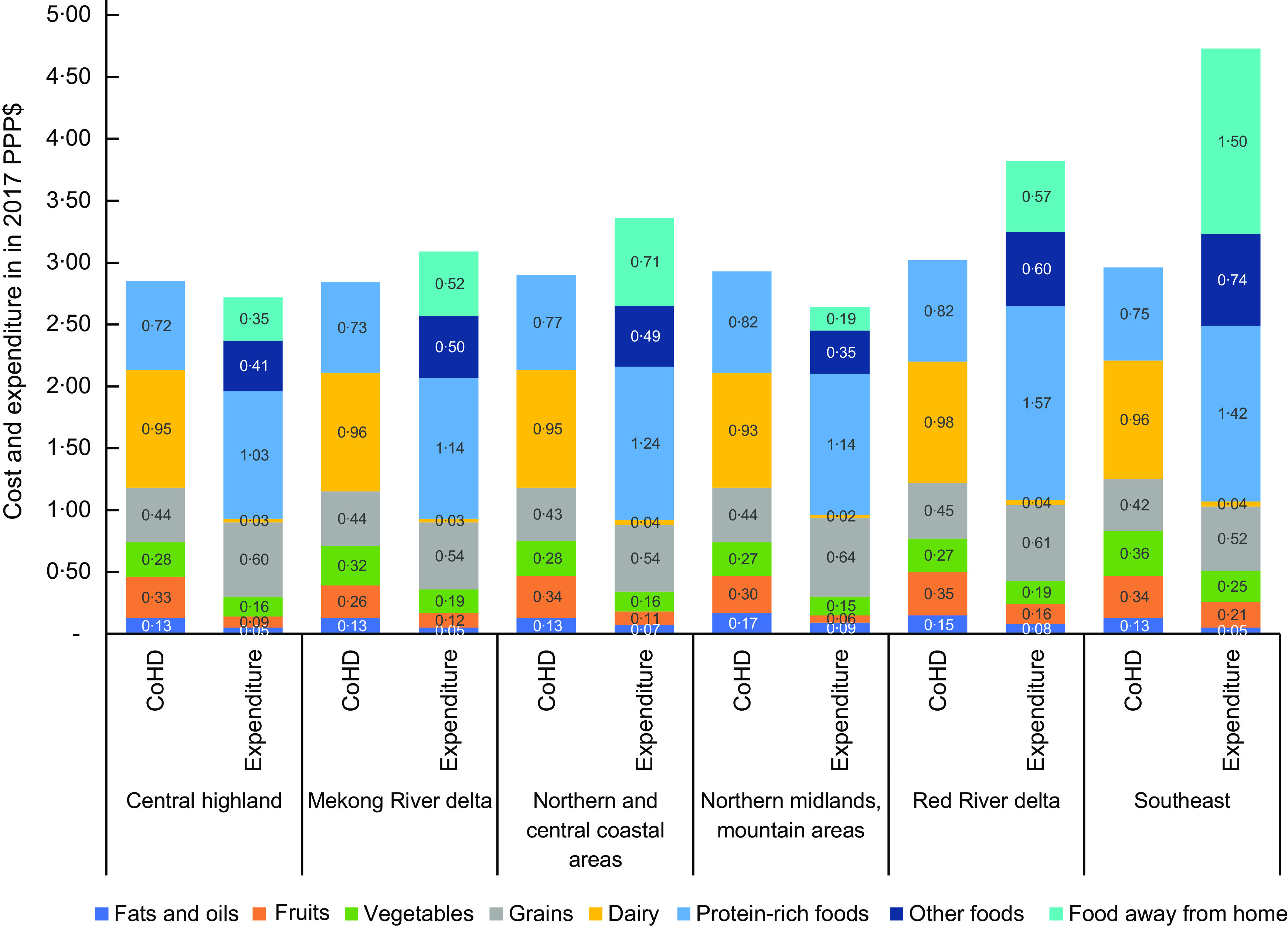
The average cost of healthy diets and median daily total food expenditure (per AFE) for each food group by region. CoHD, Cost of a Healthy Diet; AFE, adult female equivalent

The total CoHD was less than per capita food expenditures for all but the lowest income quintile in Vietnam (Table 2). In general, across regions, expenditure on grains and protein-rich foods was more than the amount needed to meet the 2016–2020 Vietnamese FBDG, whereas expenditure on other food groups, including vegetables, fruits, fats and oils, and dairy, was below the amount needed to meet the FBDG. Particularly, spending on dairy was less than $0·1 per d, much lower than the amount needed to meet the dairy recommendation at approximately $1·0 per d. Spending on other discretionary foods, such as sugary and salty foods, coffee, alcoholic beverages, and other beverages, as well as food away from home, accounted for a large portion of the total food expenditure, especially in the Southeast (Fig. 5).

**Table 2:** The cost of healthy diets as a share of mean daily total food expenditure per an adult female equivalent (AFE) by region and income level*

	2016		2018		2020		Average†	
	Median	IQR	Median	IQR	Median	IQR	Median	IQR
National average	84·6	59·0–120·3	78·0	55·0–111·0	66·1	46·6–94·1	75·6	52·8–109·4
Region								
Northern midlands, mountain areas	95·9	62·2–138·8	98·1	67·5–142·6	85·4	57·9–127·7	93·5	62·3–136·4
Red River delta	80·1	59·6–109·6	73·4	52·3–102·0	60·6	44·2–81·6	70·4	50·9–96·8
Northern and central coastal areas	86·2	61·0–122·0	76·2	55·1–107·5	64·6	45·8–90·0	74·9	53·1–107·2
Central highland	95·2	62·7–150·0	93·2	63·4–135·7	76·8	51·1–116·7	87·9	59·1–133·0
Southeast	62·1	45·3–89·4	57·7	40·8–81·1	49·1	35·9–69·1	56·2	40·0–80·1
Mekong River delta	88·5	64·5–120·4	79·5	57·7–109·4	69·1	51·0–95·7	79·3	56·6–109·2
Income level								
High income	52·0	38·3–70·8	48·7	35·6–66·7	43·4	32·7–60·0	48·7	35·6–66·7
Upper middle income	68·2	53·2–90·3	63·1	47·9–84·8	55·3	42·4–73·3	63·1	47·9–84·8
Middle income	83·2	63·9–107·9	75·0	56·6–99·6	65·1	49·9–85·4	75·0	56·6–99·6
Lower middle income	102·1	78·3–132·3	90·0	67·7–118·9	77·1	58·3–102·5	90·0	67·7–118·9
Low income	138·1	101·8–186·3	123·7	88·2–169·6	106·8	75·7–150·0	123·7	88·2–169·6

* Data are presented in median and interquartile range.

† Average from 2016 to 2020.

At the national level, affordability of healthy diets relative to a standard of total food expenditure has improved from 2016 to 2020, as shown by a reduction in the share of the CoHD to total food expenditure. A similar trend was observed across regions, except for the Northern midlands and mountain areas, where the share of CoHD to total food expenditure increased from 2016 to 2018, before decreasing from 2018 to 2020. The Southeast followed by the Red River delta showed the highest affordability of healthy diets as a share of typical daily food expenditure, while the Northern midlands and mountain areas and the Central highland showed the lowest (Table 2).

Table 3 shows that the CoHD – the least-cost healthy diet recommended by the national FBDG was not affordable for 68·4 % of low-income households, while almost all people from high-income, upper-middle-income and middle-income households were able to afford it. There was a dramatic decline in the number of people who cannot afford healthy diets in lower-middle-income households, where 54·1 % of lower-middle-income households could not afford the CoHD in 2016, but only 0·1 % could not afford it in 2020. Over 36 % of the population residing in Northern midlands and mountain areas could not afford the CoHD, while only 5·6 % of people from the Southeast could not afford it.

**Table 3:** Percentage of people who cannot afford healthy diets, by region and income level*

	2016	2018	2020	Average†
Average‡	28·9	19·6	9·9	20·0
Region				
Northern midlands and mountain areas	42·1	38·3	25·9	36·3
Red River delta	22·1	11·6	2·8	12·2
Northern and central coastal areas	31·9	21·0	10·2	21·8
Central highland	37·2	31·0	17·7	28·4
Southeast	7·2	6·2	1·6	5·6
Mekong River delta	26·9	13·6	5·1	15·7
Income level				
High income	0·0	0·0	0·0	0·0
Upper middle income	0·0	0·0	0·0	0·0
Middle income	2·7	0·0	0·0	3·0
Lower middle income	54·1	30·6	0·1	28·6
Low income	87·8	67·3	49·2	68·4

* Calculations by comparing CoHD to mean daily per capita income multiplied by the quintile average food shares.

† Average from 2016 to 2020.

‡ Average of all income levels and all regions.

**Fig. 6:**
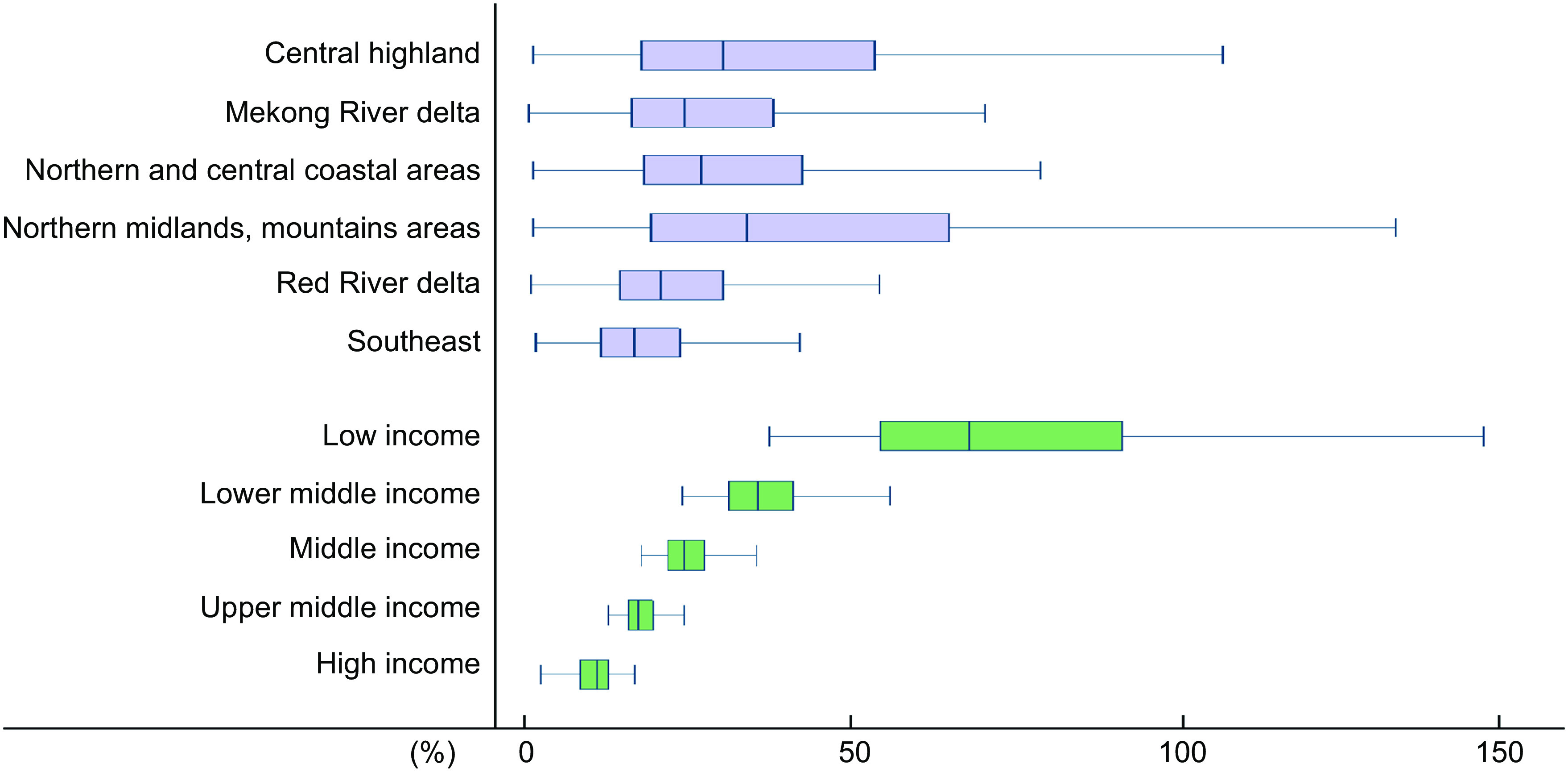
The average cost of healthy diets as a proportion of mean daily per capita income by region and income level. The size of the box shows the IQR. The bottom and top rules illustrate the fifth and fifty-nine percentiles, respectively. The vertical bar rule inside the box is the median value for the region or income level

As shown in Fig. 6, across regions, the affordability of healthy diets, as a percentage of mean daily per capita income, was the lowest in the Northern midlands and mountain areas (34·0 %; IQR 19·4–65·1) and the Central highland (30·3 %; IQR 17·9–53·7). In contrast, the highest affordability was observed in the Southeast (16·5 %; IQR 11·4–23·7) and the Red River delta (20·8 %; IQR 14·5–30·4). The affordability of healthy diets, as a percentage of mean daily per capita income, was 10·8 % (IQR 8·5–12·7) in high-income households, 17·3 % (IQR 15·7–19·4) in upper-middle-income households, 24·2 % (IQR 21·7–27·4) in middle-income households, 35·7 % (IQR 31·1–41·1) in lower-middle-income households and 68·1 % (IQR 54·5–91·7) in low-income households.

## Discussion

Using the national average CPI food price data from January 2016 to December 2020, we estimated that the national minimum cost to meet the healthy diets recommended by the 2016–2020 Vietnamese FBDG was approximately $3·08 (2017 PPP) per person per d (the average between 2016 and 2020). Our estimation is slightly lower than the estimate for Vietnam from the global analysis by Herforth *et al.*, of $3·59 (2017 PPP)^(18)^, which can be partially explained by the difference in the timescale and the difference in the food price databases. Regardless, using either figure, the cost of healthy diets in Vietnam is more expensive than the updated international poverty line of $2·15^(27)^ and the Vietnamese poverty line of $2·99 in 2017 PPP (23 300 VND per d) for rural area (Decision No. 59/2015/QD-TTg for 2016–2020 period). The affordability of healthy diets has improved from 2016 to 2020. Overall, approximately 10 % of households in 2020 were unable to afford a healthy diet at the national level. This indicates that in Vietnam, cost and affordability may not be the primary barriers to consuming a healthy diet, although we have not factored in cross-price elasticities nor the hidden costs of time, energy, and other resources in procuring and preparing food. Nonetheless, the challenge seems to be more for the poor. The number of people who cannot afford healthy diets remains high among households in the lowest income quintile.

In our study, an average household can indeed afford a healthy diet but over-consumes staples and protein-rich foods, while under-consuming dairy, fruit, and to a lesser degree vegetables. Vegetables and fruits, protein-rich foods, and dairy constituted the majority of the CoHD (approximately 80 %) in all regions. Globally, agricultural production already ensures sufficient calories for the world’s population; however, much of this production consists of energy-dense staple foods and insufficient volume and diversity of non-staple foods^(28)^. In Vietnam, the composition of the food basket has been changing remarkably, and the demand for dietary diversity has been increasing due to the country’s economic growth over the last decade^(29)^. This has led to calls for effective interventions to shift the local food systems towards the production of more diverse nutritious foods. Our findings imply that food policies should move from meeting energy needs to meeting dietary recommendations. This goal can only be achieved if diverse, nutrient-rich foods are more accessible and affordable. Thus, policies governing agriculture, marketing and trade should be adapted to address this matter along the national food supply chain. These food policies should focus on several aspects: low productivity and inadequate diversification in food production; high levels of pre-harvest and post-harvest loss in quality and quantity of agricultural products; and insufficient market infrastructure, since these were identified as key factors affecting the cost of nutritious foods and the affordability of healthy diets^(30)^.

Vegetables and fruits also had significant seasonal cost variations in our analysis. The cost of fruits and vegetables tended to decrease from May to July and increase from November to February, which may be explained by their availability during the peak season. Another study conducted in Northern Vietnam also found that the peak availability of fruits is during the hot-humid season and that availability is lowest in the cold-humid season^(31)^. Other studies also showed that highly perishable foods like fruits and vegetables are more sensitive to seasonality than foods with a longer shelf life, such as grains^(32,33)^. Seasonal variability in food prices may have consequences for food price volatility and availability of food products and further impact food security, nutrition, and health^(34)^. This is particularly problematic for Vietnamese people, who prefer fresh foods, especially vegetables and fruits from the fields or wet markets, rather than frozen or canned products^(35)^. Thus, to maintain diet quality throughout the year, transport and storage systems across the country should develop strategies robust to seasonality that can supply vegetables and fruits in consistent quantities.

The Vietnamese dietary pattern is dominated by grains (of which white rice is the primary source), which provide approximately 70 % of total dietary energy intake^(10)^. Given this dominance, it is unsurprising to see that the Vietnamese population overspends on these foods; and similar observations are found elsewhere in the literature, especially for Asian countries^(19,30)^. Although rice demand is decreasing in both rural and urban households in Vietnam^(29)^, it still remains a key food item, as the price of rice affects directly household energy intake, and keeping rice available at a reasonable price allows the poorest to more easily diversify out of the staple and into more nutritious foods^(36)^.

The Vietnamese FBDG define protein-rich foods as a food group, which includes both plant- and animal-source foods. Plant-source protein-rich foods are usually selected as the least-cost items in that food group. Expenditure data show that Vietnamese people tend to spend more on this food group than the least cost; purchasing more than the absolute lowest cost on protein-rich foods reflects the actual consumption of animal-source protein-rich foods, which are more expensive than the least-cost plant-source protein-rich foods.

The CoHD based on the Vietnamese FBDG fulfils more than 80 % of nutrient requirements on average (calculated based on the RDA for Vietnamese, see online supplementary material, Supplemental Table S4) for both males and females (see online supplementary material, Supplemental Table S5). It does not always meet requirements for Fe (for female), Zn, vitamin A and vitamin B_12_. These are nutrients particularly well provided by animal-source foods; therefore least-cost diets may need to include animal-source foods to more consistently meet all micronutrient requirements, although this would increase the costs^(18)^.

Dairy is the most expensive component of healthy diets. Although Vietnam’s dairy industry has developed in recent times and contributed significantly to the local needs, retail milk price remains high in Vietnam^(37)^. Current expenditures on dairy are close to zero, as observed previously^(10)^. There may be multiple reasons for the low consumption of dairy. Vietnam is one of the countries in Asia with the highest prevalence of lactose malabsorption, which might cause digestive discomfort following consumption and reinforce dairy avoidance in the population^(38)^. There is also a growing body of evidence showing the impacts of dairy products on the environment, which might be another potential reason for low dairy consumption^(39)^. However, dairy is still recommended as a food group in the Vietnamese FBDG based on its contribution to nutrient intakes, particularly Ca. Our results on unaffordability of dairy, combined with apparent low acceptability and consumption of dairy, suggest that these sociocultural and environmental factors may need to be considered to make the Vietnamese FBDG more affordable, acceptable and sustainable.

The cost and affordability of healthy diets differ significantly across regions, and the share of each food group to the total cost of healthy diets also varies by region. Regions in Vietnam are known to be different in terms of socio-economic characteristics and also expenditures on food^(40)^. The Northern midlands and mountain areas showed the lowest affordability of healthy diets. One reason may be due to issues like poverty which is more pronounced in the rural area and this part of the country^(14)^. In contrast, the Southeast and the Red River Delta areas showed the highest affordability. These two regions have the highest average incomes^(14)^ in Vietnam and consist of the largest cities in Vietnam with weighty urban population growths, food system transformations and subsequent nutrition transitions. Our previous study also showed that the diet quality of the general population varied by region, as the largest percentage of participants with higher diet quality scores were from the Red River delta^(10)^. These results further support the findings that households with higher income have access to healthier foods that may be unaffordable to households with lower income, which positively impacts their diet quality.

In general, between 2016 and 2020, healthy diets were becoming more affordable in the country, as explained by the increase in income per capita in the whole country during this period (from 2016 to 2020, income increased by 8·2 %). Although income per capita in 2020 decreased by about 1% compared with 2019 due to the impact of the COVID-19 pandemic, the poverty rate still reduced in 2020. It is the result of the implementation of social security policies by the Vietnamese Government. Nonetheless, there is still a large difference in living standards between urban and rural, the rich and the poor. We found that while the healthy diets recommended by the FBDG was affordable for all people in high-income, upper-middle-income and middle-income households, it was unaffordable for approximately 70% of low-income households, where adherence can cost up to almost 70% of their income. It should be kept in mind that the calculation of the cost is likely to be an underestimate as it does not consider individual tastes and preferences but instead simply chooses the lowest-priced items in each food group. Thus, households in the low-income class would have to spend the majority of their total income just to access healthy diets that adhere to dietary guidelines. In such circumstances, purchasing a healthy diet is infeasible. Remarkably, we also observed a dramatic decrease in the percentage of people who cannot afford healthy diets of lower-middle-income households from 2016 to 2020. Here, the role of income is more obvious than the cost since the cost of healthy diets increased only slightly during this period (after inflation adjustment), while income increased significantly. Trinh *et al.* also observed a strong correlation between energy intake and income for the poorest households, indicating that there is still room for income-based policies to fight against malnutrition in Vietnam^(41)^. Household income level affects not only energy intake but also diet quality. Another study in Vietnam showed that urban and rich households consume less staple foods such as rice and more non-staple foods such as fruits and vegetables and animal-source foods and that rural and poor households are likely to follow this dietary change when their income increases^(29)^. Thus, higher income and lower food prices are together required to make healthy diets more affordable for Vietnam’s poor population. Bell *et al.* found that other factors beyond food prices and income, such as consumer preferences, food safety, taste, convenience, and other food quality attributes, also drive food consumption patterns among Vietnamese populations^(42)^. Therefore, these factors, along with other social factors, must also be considered in order to enable people to follow the diets recommended by the national FBDG^(43)^.

This present study has some limitations. First, we chose to calculate the CoHD since this indicator obtains the lowest cost of meeting the national FBDG that are typically tailored to country-specific nutritional conditions. However, some habitually consumed foods might not fall into the CoHD baskets, and the CoHD might be likely to underestimate the cost of healthy and preferred diets^(44)^. Future research might use the modified CoHD indicator called the Food Preferences CoHD (CoHD-FP) introduced by Mahrt *et al.* to gain insight into the cost of acquiring the recommended healthy diet while considering actual dietary preferences, which has also been applied to construct nutrition-sensitive poverty lines^(44,45)^. The food consumption data conversion might cause inaccuracy in estimating individual food consumption, which can be overcome with the availability of individual dietary intake data in future studies. We were not able to measure the composition of foods consumed away from home due to the limitation of the household consumption and expenditure survey data. Parts of the data were also collected during the first wave of the COVID-19 pandemic in Vietnam (2019–2020), which may have influenced the estimates of the cost and affordability of healthy diets during this period.

In conclusion, the calculations of the cost of the current FBDG would be valuable for the development of the new 2021–2025 Vietnamese FBDG, to make them more feasible and achievable. The CoHD is a straightforward indicator, so local policymakers and researchers can apply it on available food price data to track the affordability of healthy diets on a timelier and more regular basis. This would enable regular evaluation of the ability of the local food system to deliver healthy diets and further protect food security, nutrition and health in Vietnam.

## Supporting information

Van et al. supplementary material Van et al. supplementary material

## Supplementary material

For supplementary material accompanying this paper visit https://doi.org/10.1017/S1368980023002665
