# Supplementary material for: Cost and affordability of healthy diets in Vietnam
Source: Public Health Nutr. 2023 Dec 1;27(1):e3. doi: 10.1017/S1368980023002665 (PMC10830355; doi:10.1017/S1368980023002665)
Supplement: Van et al. supplementary material [file S1368980023002665sup001.docx]

**Table S1.** The list of 25 provinces representing six regions in Vietnam, based on the General Nutrition Survey 2019-2020 sample size selection

| **Region** | **Provinces** | **Population in 2019** |
| --- | --- | --- |
| Northern midlands and mountain areas | Cao Bằng | 530,341 |
|  | Sơn La | 1,248,415 |
|  | Thái Nguyên | 1,286,751 |
|  | Bắc Giang | 1,803,950 |
| Red River delta | Hà Nội | 8,053,663 |
|  | Quảng Ninh | 1,320,324 |
|  | Hải Dương | 1,892,254 |
|  | Hưng Yên | 1,252,731 |
|  | Hà Nam | 852,800 |
| Northern and central coastal areas | Thanh Hoá | 3,640,128 |
|  | Nghệ An | 3,327,791 |
|  | Thừa Thiên Huế | 1,128,620 |
|  | Quảng Ngãi | 1,231,697 |
|  | Khánh Hoà | 1,231,107 |
| Central highland | Gia Lai | 1,513,847 |
|  | Đắk Lắk | 1,869,322 |
|  | Lâm Đồng | 1,296,906 |
| Southeast | Tây Ninh | 1,169,165 |
|  | Bình Dương | 2,426,561 |
|  | Đồng Nai | 3,097,107 |
|  | Hồ Chí Minh | 8,993,082 |
| Mekong River delta | Bến Tre | 1,288,463 |
|  | Đồng Tháp | 1,599,504 |
|  | Cần Thơ | 1,235,171 |
|  | Cà Mau | 1,194,476 |

* Source from National institute of Nutrition, Ministry of Health, Vietnam.

**Table S2.** Number of food items by food group for Consumer Prices Index food prices data

| **Food groups** | **Main food items** | **Number of food items** | |
| --- | --- | --- | --- |
|  |  | **Original CPI list** | **Final CPI list** |
| Grains | Rice, bread, noodles, potato, corn, casava | 18 | 12 |
| Protein-rich foods | Red meat, poultry, seafood, eggs, legumes and beans, and soy products | 42 | 39 |
| Vegetables | All type of vegetables and mushroom | 14 | 14 |
| Fruits | All type of fruits | 16 | 12 |
| Dairy | Milk, yogurt, cheese | 8 | 8 |
| Fats and oils | Animal fats, plant-based oil, butter, nuts, and seeds | 4 | 3 |
| Discretionary foods | Sugary and salty foods, coffee, alcoholic beverages and other beverages, and foods eating away from home | 74 | 0 |
| **Total** |  | **176** | **88** |

**Table S3.** Median food expenditure shares in high-, upper-middle-, middle-, lower-middle- and low-income households in 2016, 2018 and 2020

|  | **2016** | | **2018** | | **2020** | |
| --- | --- | --- | --- | --- | --- | --- |
| Low-income | 0.60 |  | 0.58 |  | 0.55 |  |
| Lower-middle-income | 0.41 |  | 0.39 |  | 0.39 |  |
| Middle-income | 0.34 |  | 0.32 |  | 0.32 |  |
| Upper-middle-income | 0.29 |  | 0.26 |  | 0.28 |  |
| High-income | 0.22 |  | 0.20 |  | 0.21 |  |

**Table S4.** Nutrient reference values for a representative Vietnamese adult^*^

|  | Unit | Male^†^ | Female^‡^ |
| --- | --- | --- | --- |
| Energy | kcal | 2,570 | 2,050 |
| Macronutrients |  |  |  |
| Protein | g | 69.0 | 60.0 |
| Carbohydrate | g | 370.0-400.0 | 320.0-360.0 |
| Dietary fibre | g | 38.0 | 25.0 |
| Total fat | g | 57.0-71.0 | 46.0-57.0 |
| Micronutrients |  |  |  |
| Calcium | mg | 800.0 | 866.7 |
| Potassium^§^ | mg | 2,500 | 2,000 |
| Magnesium | mg | 340.0 | 270.0 |
| Iron^ǁ^ | mg | 11.9 | 26.1 |
| Zinc^¶^ | mg | 20.0 | 16.0 |
| Thiamine | mg | 1.3 | 1.1 |
| Riboflavin | mg | 1.5 | 1.2 |
| Niacin | mg | 16.0 | 14.0 |
| Vitamin A | µg RAE | 850 | 650 |
| Vitamin E (alpha-tocopherol)^§^ | mg | 6.5 | 6.0 |
| Vitamin C | mg | 100.0 | 100.0 |
| Vitamin B5 | mg | 5.0 | 5.0 |
| Vitamin B6 | µg | 1.3 | 1.3 |
| Folate | µg | 400.0 | 400.0 |
| Vitamin B12 | µg | 2.4 | 2.4 |

RAE, Retinol Activity Equivalent.

* Values are Recommended Dietary Allowances (RDAs) or Adequate Intake (AI) adapted from the 2016 Vietnamese recommended dietary allowances book^(41)^.

† Values shown are for a Vietnamese man aged 20-29 years.

‡ Values shown are for a Vietnamese non-pregnant, non-lactating woman aged 20-29 years.

§ Values are Adequate Intake (AI).

ǁ Based on iron bioavailability of 10%.

¶ Based on zinc low absorption.

**Table S5.** Percent of nutrient recommendations met by the Cost of a Healthy Diet (CoHD) pattern according to mean adequacy ratio

| Energy and nutrient intakes^*^ | Average values of a CoHD pattern^†^ | NAR-Male | NAR-Female |
| --- | --- | --- | --- |
| Energy | 2019.5 | 0.79 | 0.99 |
| Macronutrients (g) |  |  |  |
| Protein | 75.2 | 1.00 | 1.00 |
| Carbohydrate | 313.4 | 0.85 | 0,98 |
| Dietary fibre | 12.4 | 0.33 | 0.50 |
| Total fat | 51.7 | 0.91 | 1.00 |
| Saturated fat | 12.2 | - | - |
| Monounsaturated fat | 16.8 | - | - |
| Polyunsaturated fat | 15.9 | - | - |
| Micronutrients |  |  |  |
| Calcium (mg) | 695.3 | 0.87 | 0.80 |
| Potassium (mg) | 4,145 | 1.00 | 1.00 |
| Sodium (mg) | 1,260 | - | - |
| Magnesium (mg) | 538 | 1.00 | 1.00 |
| Iron (mg) | 15.9 | 1.34 | 0.61 |
| Zinc (mg) | 10.6 | 0.53 | 0.66 |
| Thiamine (mg) | 1.2 | 0.92 | 1.00 |
| Riboflavin (mg) | 1.5 | 1.00 | 1.00 |
| Niacin (mg) | 9.7 | 0.61 | 0.69 |
| Vitamin A (µg RAE) | 335.5 | 0.39 | 0.52 |
| Vitamin E (mg) | 4.2 | 0.65 | 0.70 |
| Vitamin C (mg) | 111.9 | 1.00 | 1.00 |
| Vitamin B5 (mg) | 7.4 | 1.00 | 1.00 |
| Vitamin B6 (µg) | 1.7 | 1.00 | 1.00 |
| Folate (µg) | 1,111 | 1.00 | 1.00 |
| Vitamin B12 (µg) | 1.7 | 0.71 | 0.71 |
| Mean Adequacy Ratio (MAR)^*^ |  | **0.83** | **0.86** |

CoHD, Cost of a Healthy Diet. NAR, Nutrient Adequacy Ratio. RAE, Retinol Activity Equivalent. MAR, Mean Adequacy Ratio.

† Nutrient values are analysed based on the 2019 Vietnamese Food Composition Table^(42)^.

* Data is the average of 60 CoHD patterns, from January 2016 to December 2002.

‡ Values are analysed across 20 nutrients with reference values adapted from Table S2.
